# Mine fire emergency path planning based on hybrid strategy improved WOA algorithm

**DOI:** 10.1371/journal.pone.0323789

**Published:** 2025-05-28

**Authors:** Yun Qi, Kaiwang Yu, Xunping Li, Wei Wang, Xinchao Cui, Chenhao Bai

**Affiliations:** 1 College of Mining and Coal, Inner Mongolia University of Science and Technology, Baotou, Inner Mongolia, China; 2 College of Coal Engineering, Shanxi Datong University, Datong, Shanxi, China; Hanshan Normal University, CHINA

## Abstract

To address the challenge of personnel evacuation during mine fires, an enhanced Whale Optimization Algorithm (WOA) incorporating a hybrid strategy inspired by the intelligent behavior of marine life is proposed and applied to mine escape route planning. Initially, to overcome the limitations of the original WOA—such as poor optimization accuracy, susceptibility to local optima, and slow convergence—five improvement strategies are introduced: Sobol sequence for population initialization, nonlinear time-varying factors, adaptive weighting, stochastic learning, and Cauchy mutation. These enhancements are compared against single-strategy improved WOAs.Subsequently, path planning simulations were conducted using several extracted algorithms and grid-based methods. The results demonstrate that the optimal path length achieved by the Multi-Strategy WOA (MSWOA) is 41.7% shorter than that of the standard WOA, 42.3% shorter than WOA-1, and 48.5% shorter than PSO for the shortest path. Additionally, the average path length of MSWOA is 32.2% shorter than WOA, 40.5% shorter than WOA-1, and 41.4% shorter than PSO. The MSWOA algorithm generates the shortest and smoothest path among the tested methods.Based on the analysis of the path graph and iteration frequency graph, it is recommended to apply the MSWOA algorithm to path planning experiments. The findings indicate that the WOA with the five integrated strategies significantly enhances optimization accuracy and convergence speed, making it a robust solution for mine evacuation route planning.

The occurrence of mine disasters [1] often results in significant loss of life and property, making it critically important to develop effective countermeasures to mitigate such disasters. Mine fire disasters, in particular, pose a severe threat to safe and efficient production [2]. Traditional route planning methods rely solely on pre-established emergency escape routes, which lack flexibility and fail to adapt to the actual conditions during a fire. Currently, intelligent bio-inspired algorithms [3] are being utilized for emergency path planning in the aftermath of a fire. The application of such emergency path planning in the mining industry is also one of the key challenges attracting significant attention. With the rapid development of coal mine productivity, mine fires have become a serious hazard, endangering both lives and property [4]. Therefore, identifying the optimal escape path within the roadway after a fire plays a crucial role in minimizing the loss of life and property caused by mine fire accidents.

In previous research on path planning, traditional graph algorithms, such as Dijkstra’s algorithm and Floyd’s algorithm, have shown significant limitations. In complex information environments, the computational load increases dramatically, often exponentially. This exponential growth in computation undoubtedly raises both the difficulty and time cost of solving such problems. In recent years, with the continuous advancement of computer technology, sensing technology, and control technology, intelligent optimization algorithms have emerged and gained widespread application in the field of path planning. These algorithms are often inspired by the behaviors of biological organisms, with common examples including the Ant Colony Optimization (ACO) algorithm and the Particle Swarm Optimization (PSO) algorithm.

Many scholars have carried out in-depth research on the problem of optimal route planning, which is the essence of emergency evacuation path. For example, Zhang Yingfei et al. [5] proposed a linear cellular automata model with ripple diffusion as the core to explore the crowd evacuation path, and then obtained an emergency path that could shorten the evacuation time. Cao Xianghong [6] et al. improved the gravity function to generate the shortest evacuation route while staying away from the ignition point. Wang Tao [7] et al took the risk value suffered by evacuees as the key condition for path search, and applied the improved Dijkstra algorithm to finally obtain the evacuation path with the least personnel damage. Ye Jihong et al. [8] incorporated the toxicity load value of the evacuation path into the heuristic function of the ant colony algorithm, effectively reducing the impact of toxic gas on the evacuees. Zhang Haipeng et al. [9] adopted the Floyd algorithm and combined it with the hydraulic model to successfully obtain the shortest evacuation route in the fire scenario. Yang Qian [10] et al. used genetic algorithm to implement two-stage planning for emergency vehicle routes of sudden natural disasters, and used hierarchical analysis of hierarchical demand degree of legal empowerment to build an efficient material distribution model. Jia Jinzhang [11] et al., in order to solve the evacuation path of personnel more efficiently, proposed a hybrid gene-ant colony algorithm when a fire broke out. Luo Wenguan et al. [12] established an emergency UAV path planning model and proposed a cuckoo search algorithm for planning high-quality UAV rescue routes based on reinforcement learning. Lin Yong [13] et al. improved the path planning of multi-objective particle swarm optimization algorithm on the basis of coding correction in response to the distribution of emergency materials for earthquake disasters. By improving the algorithm, Zhang Huajun et al. [14] carried out optimal path planning in the context of cruise fire spread, and determined the optimal path by analyzing risk indicators such as fire gas. He Mengnan et al. [15] proposed a Global shortest path (GSPCA) optimization algorithm based on cellular automata, which can shorten the shortest path by 12.39% on average and flexibly choose solution methods according to terrain features. Cao Xianghong et al. [16] proposed Dijkstra-ACO hybrid path dynamic algorithm to obtain the optimal path. Liu Huilin et al. [17] used the Gaussian gas diffusion model to calculate the toxicity load value of injury indicators such as death probability of each evacuation section, so as to screen out the best route. Zhang Mingkong et al. [18] proposed a coevolutionary path optimization method, Ripple Diffusion algorithm (RSA), and adopted CEPO to optimize personnel evacuation paths. In summary, although some achievements have been achieved in emergency path planning through improved algorithms and the integration of different algorithms, there are still many problems. On the one hand, the traditional map search algorithm has its own defects. With the increasing of environmental information, its computational complexity will increase geometrically. On the other hand, general intelligent algorithm, as a new way of path planning, has high requirements on computer performance, its operation process is more complicated, the universality of the algorithm is relatively poor, and it is easy to fall into the dilemma of local optimization. Therefore, general intelligent algorithm has high requirements on computer performance in practical application, and the operation process is also relatively lengthy, and the universality of the algorithm is not good.

Therefore, the author proposes a mixed-strategy-based improved Whale Optimization Algorithm (MSWOA) incorporating five mixed strategies. This enhanced algorithm addresses issues such as low precision, local optima, and slow convergence speed, enabling it to achieve the optimal path more efficiently. The algorithm is applied to path planning, where an appropriate fitness function is selected for individual evaluation. It is then compared with mainstream algorithms and single-strategy improved whale optimization algorithms. The results demonstrate that MSWOA exhibits strong global search capabilities, excellent optimization performance, and fast convergence. Simulation results further confirm the superiority of the MSWOA algorithm, offering a novel approach for mine fire personnel escape and emergency path planning.

## 1 Whale optimization algorithm

### 1.1 Algorithm introduction

The Whale Optimization Algorithm (WOA) is a novel swarm intelligence optimization algorithm inspired by the foraging behavior of whale populations. It was proposed by Mirjalili et al. [19] in 2016 to achieve optimal solutions for target problems. The algorithm establishes theoretical models based on behaviors such as searching, encircling, and hunting. Compared to other optimization algorithms, WOA primarily consists of three stages: hunting prey, encircling prey, and spiral bubble-net hunting. Its key advantage lies in the unique search mechanism, which is developed by simulating the hunting behavior of humpback whales. Additionally, WOA features fewer parameters and is relatively easy to understand, making it both efficient and accessible.

### 1.2 Hunt for prey

The behavior of each whale participating in the hunt represents a finite solution x in the whale optimization algorithm. In the first stage of hunting prey, the whales’ experience is based on the position information of each other in the group, updating the position of the next generation, and through random search, based on the random walk mechanism during the group hunting, so that the algorithm has excellent performance in the overall search. According to the t search, At that time |A|(1 , The whale updates the search behavior for t + z1, as follows:


$$x(t+1)=x_{\text{rand}}-A\cdot D_{\text{rand}}($$ (1)

among $D_{\text{rand}}=\left|c\cdot x_{\text{rand}}-x(t)\right|$, $x_{\text{rand}}$ to hunt any whale location in the herd,x(t) is the current position, t is the current number of iterations, and:


A=2ar−a( (2)

c=2r( (3)

among r∈rand[0,1],a=2−2t/T,T is the number of iterations. As the number of iterations increases,a it’s a linear decline, Correspondingly, it gradually decreases A witha the decline, At that time |A|≤1, The algorithm moves from the prey search phase to the second phase to surround the prey.

### 1.3 Round up prey

As the number of iterations increases,|A| reduce to 1. The algorithm moves into its second phase, which revolves around the prey. Since the location of prey is fuzzy during the initial search, it is assumed that the fit degree value and the optimal solution in the whale group at this time are the WOA algorithm for the location of prey or the location of the target prey. Other whales adjust their positions by gradually shrinking according to the current optimal location information when approaching prey and determining the target location of prey.


$$x(t+1)=x^{*}(t)-A\cdot D_{1}($$ (4)

among, $D_{1}=\left|c\cdot x^{*}(t)-x(t)\right|$, $x^{*}(t)$ the optimal solution for each iteration. When betweenA∈[−1,1] random values,x(t+1) update x(t) from i$x^{*}(t)$ teration to iteration, Gradually approaching the surrounding optimal solution.

### 1.4 Spiral bubble net hunting

The best whale individuals are unique in their hunting strategies by spiraling their prey close to the surface of the ocean and capturing it in the best possible way while surrounding it. The mathematical representation of this rare hunting behavior is as follows:


$$x(t+1)=D_{2}{\text{e}}^{bl}\cos(2\text{π}l)+x^{*}(t)($$ (5)

Where, b is the constant coefficient of the spiral equation, take b=1; $D_{2}=\left|x^{*}(t)-x(t)\right|$ Represents the distance between the search individual and the current optimal solution individual; l Is [0,1]a random number inside.

In order to simulate the hunting behavior of whales which can simultaneously carry out encircling contraction and spiral bubble net hunting, the position updating strategy was selected by setting random probability. When the probability is p≥0.5 random, The whales update their hunting position by spiraling around in a bubble net, getting closer to their prey by blowing bubbles, When the probability is p(0.5 random, The whales update their encircling hunting position based on the position of the current optimal solution individual. In the iterative process of approaching the target |A| It |A| keeps decreasing, At that time |A|=0, The whale optimization algorithm has found the theoretical optimal solution. The overall hunting mechanism is described as follows:


$$x(t+1)=\left\{ \begin{array}{c} x^{*}(t)-A\cdot D_{1},P(0.5\\ D_{2}{\text{e}}^{bl}\cos(2\text{π}l)+x^{*}(t),p\geq 0.5 \end{array}\right.($$ (6)

## 2 Hybrid strategy improved whale optimization algorithm

### 2.1 Improved algorithm generalization

In this paper, the Sobol sequence is employed to initialize the whale population, enhancing the quality of the initial solutions. The algorithm’s global exploration and local exploitation capabilities are improved through a strategy that balances nonlinear time-varying factors and adaptive weights. Additionally, a Cauchy mutation operator is introduced to help the algorithm escape from local optima. Building on these four improvements, a hybrid-strategy improved Whale Optimization Algorithm (WOA) is proposed [20], which integrates the aforementioned enhancements to achieve superior performance.

### 2.2 Sobol sequence initializes the population

**Fig 1:**
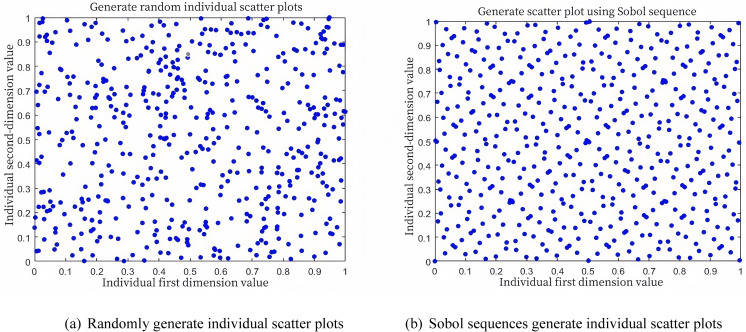
Distribution of individuals of generated by random method and Sobol sequence.

The distribution of initial solutions in the solution space significantly impacts the convergence speed and optimization accuracy of the algorithm. A uniform distribution of initial solutions helps enhance the algorithm’s performance. The traditional Whale Optimization Algorithm (WOA) initializes the population randomly, which often leads to an uneven distribution of the initial population. In this paper, the Sobol sequence is utilized to initialize the population. The Sobol sequence is a quasi-random sequence that employs base-2 radical inversion in each dimension of the population, with each dimension having its own unique matrix. This approach generates non-repetitive and relatively uniform points.To compare the spatial distribution of random numbers generated by the traditional WOA initialization method with those generated by the Sobol sequence, a two-dimensional random number distribution diagram was created for a population size of 500 within the range [0, 1]. As shown in Fig 1, the initial population generated using the Sobol sequence exhibits a more uniform distribution and provides more comprehensive coverage of the solution space.

### 2.3 Nonlinear time-varying factors and adaptive weights

**Fig 2:**
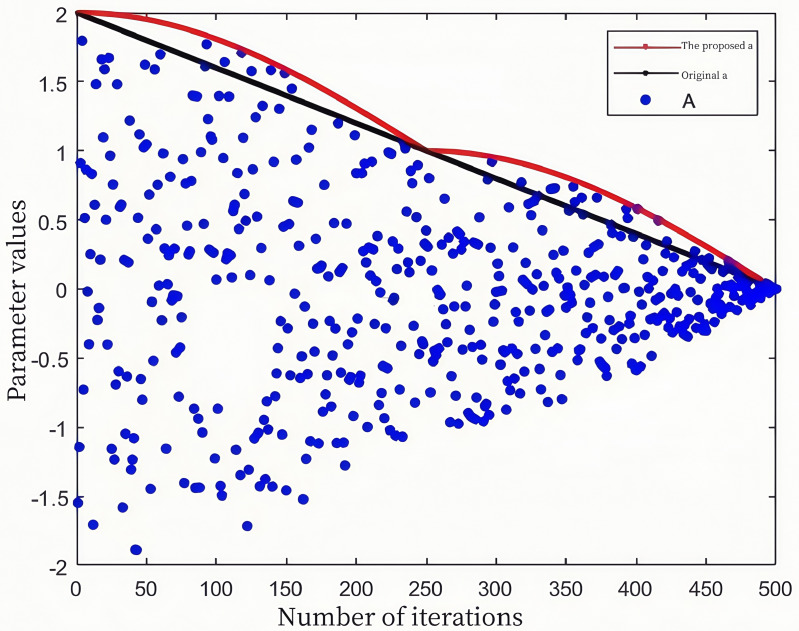
Distribution of convergence factor and parameter *A.*


Parameter A, primarily influenced by the convergence factor, is a critical parameter that regulates global exploration and local exploitation in the Whale Optimization Algorithm (WOA). As illustrated in Fig 2, the convergence factor decreases linearly as the number of iterations increases. This linear decrease can lead to slow convergence during the later stages of the hunting process and may result in incomplete exploration of the prey’s location in the early stages. To address the limitations of the convergence factor, this paper proposes a piecewise nonlinear convergence factor, defined as follows:

$$\left\{ \begin{array}{c} a=1+\cos(\text{π}\times \frac{t}{T}),t\leq 0.5T\\ a=\cos\left(\text{π}\times \frac{t-0.5T}{T}\right),t(0.5 \end{array}\right.($$ (7)

Fig 2 illustrates the distribution of the convergence factor and parameter A over 500 iterations. As shown in the diagram, the proposed convergence factor is larger than the traditional convergence factor during both the early and late stages of iterations. This enhancement strengthens the algorithm’s global exploration capability in the early stage and its local exploitation capability in the later stage, while maintaining the original transition point between global exploration and local exploitation.

In the process of hunting, the adaptive weight strategy is proposed, taking into account the impact of prey targets on whale group position update, as follows:


$$\omega (t)=\frac{2}{\text{π}}\times \tan\frac{t}{T}($$ (8)

The location update formula becomes:


$$x(t+1)=x_{\text{rand}}(t)\times \omega -A\times D_{\text{rand}},p(0.5(|A|(1($$ (9)

$$x(t+1)=x^{*}(t)\times \omega -A\times D_{1},P(0.5(|A|\leq 1($$ (10)

$$x(t+1)=D_{2}{\text{e}}^{bl}\cos\left(\text{2πL}\right)+x^{*}(t)\times (1-\omega ),p\geq 0.5($$ (11)

As the number of adaptive weight changes increases, a nonlinear upward trend is observed. In the early iterations, the weight is small, and the target is relatively vague, making position updates less impactful. However, in the middle and later stages of the algorithm, the target becomes clearer, and the weight gradually increases with the number of iterations. This increasing weight enhances the attractiveness of the target to individual whales, enabling them to locate the target solution more quickly and accurately. This mechanism effectively strengthens the algorithm’s optimization performance, accelerates its convergence rate, and improves its stability. By adopting a global optimization approach, the algorithm also enhances its local exploitation capability.

### 2.4 Stochastic learning strategy

Whales possess the ability to communicate, navigate, locate, and hunt in water. Inspired by this behavior, a stochastic learning strategy is introduced into the teaching and learning-based optimization algorithm [21] to simulate the collaborative learning and communication among whales. This strategy allows individuals to optimize their positions by learning from high-performing individuals within the population. In contrast, the traditional Whale Optimization Algorithm (WOA) relies solely on random individuals in the population to guide position updates during global search, which limits its overall optimization capability.For an individual x, different individuals (xp) are selected from the population, and their fitness values are compared to identify better-performing individuals. The position of individual x is then adjusted by learning from these better-performing individuals. This process involves selecting different individuals from the population, comparing their fitness values, and updating positions based on the insights gained from superior individuals. This approach enhances the algorithm’s ability to seek optimal solutions and improves its overall performance.


$$x_{new}=\left\{ \begin{array}{c} x+\text{rand}(0,1)\times (x-x_{p}),f(x_{p})(f(x)\\ x+\text{rand}(0,1)\times (x_{p}-x),f(x_{p})(f(x) \end{array}\right.($$ (12)

Among them, The random number between the learning factors rand(0,1) and(0,1) reflects the difference in each individual’s learning ability. After studying, if $f(x_{new})(f(x)$, Then the population accepts the new individual xnew and replaces individual x, otherwise the population rejects the inferior individual xnew. Stochastic learning of individuals will provide more information to individuals, enhance the information sharing ability of populations, increase population diversity, and effectively improve the global optimization performance of the algorithm.

### 2.5 Cauchy mutation strategy

To address the issue of the traditional Whale Optimization Algorithm (WOA) being prone to falling into local optima, a Cauchy mutation strategy is introduced based on the Cauchy distribution. This strategy applies Cauchy disturbance to the current best individual. When an individual becomes trapped in a local optimum, the Cauchy operator can generate a larger step size, enabling the algorithm to escape the local optimum. Conversely, it can also produce a smaller step size, thereby accelerating the search for the optimal solution. To implement this, the following Cauchy variant is used in this paper:


$$x_{new}^{*}(t)=x^{*}(t)\times (1+\text{cauchy}(0,1))($$ (13)

Among them,$x_{new}^{*}(t)$ is the new value obtained from the current optimal value by Cauchy perturbation, cauchy(0,1) is a Cauchy operator, and the standard Cauchy distribution function formula is as follows:


$$f(x)=\frac{1}{\text{π}\times (x^{2}+1)},x\in (-\infty ,+\infty )($$ (14)

From the mathematical theory, it can be known that the extension of the two ends of the Cauchy distribution function can produce random numbers away from the origin, and the disturbance ability of the individual whale is very strong, and the whale will be able to quickly avoid the local trap after the disturbance. At the same time, it can reduce the time spent by individuals in the search space, so as to speed up the convergence of the algorithm, when the highest peak value of the Cauchy distribution is low compared with other functions.

## 3 Algorithm implementation process

The flow of the MSWOA algorithm is described as follows. (1) initialization algorithm related parameters: population size N, spatial dimensiondim, the searchable space of the population [ub,lb], iteration coefficient T。

**Fig 3:**
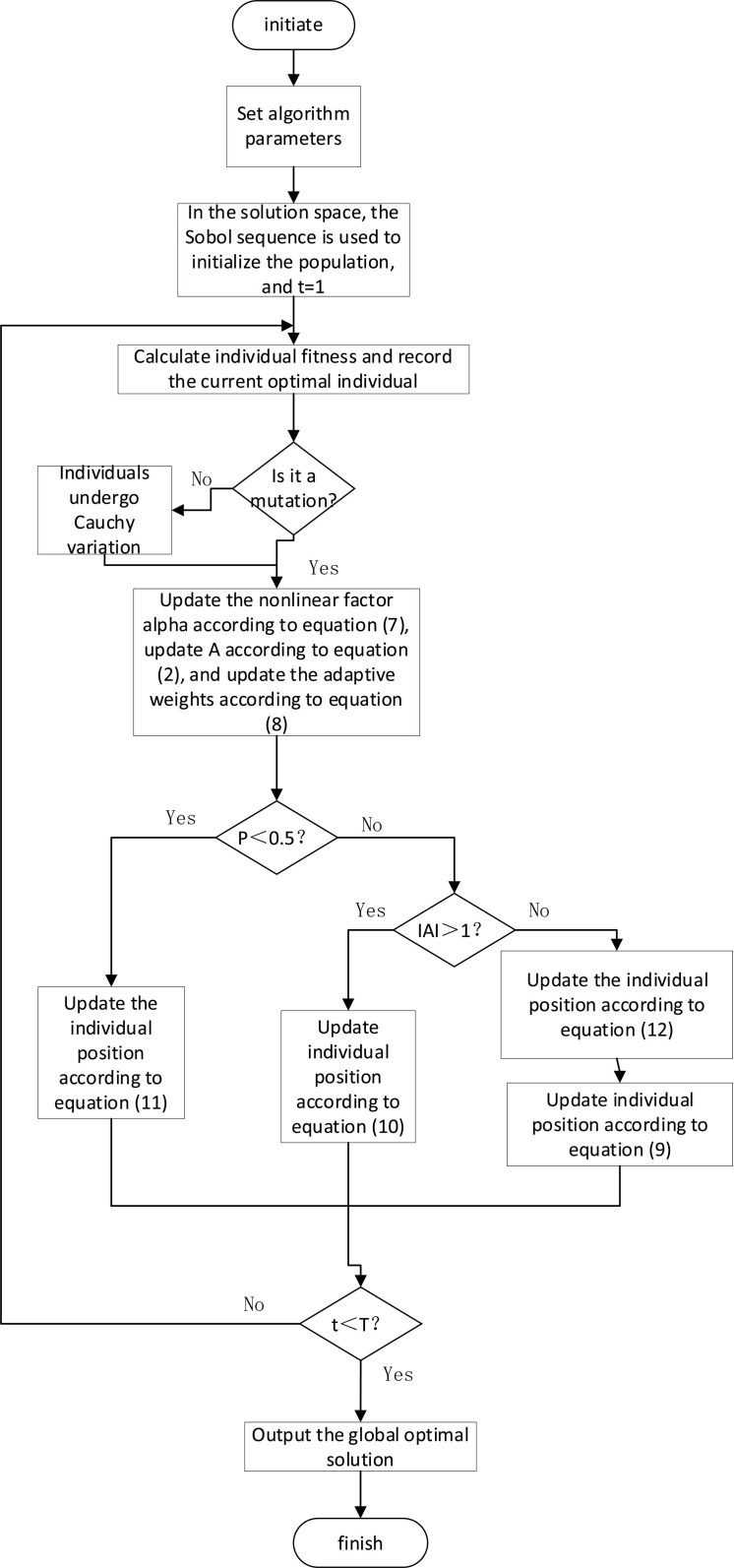
Algorithm flow chart.

(2) Sobol sequence strategy was used to initialize the population position.

(3) By calculating individual fitness, the optimal fitness and the corresponding optimal fitness are found. The optimal fitness value is constant for 10 consecutive iterations. Then formula (13) operates the variation of the optimal individual.

(4) Update parameters according to equation (7) α, Then update *A* according to formula (2), Update ω parameters according to equation (8), And updatec(p parameters at the same time/

(5) At that time p(0.5,|A|(1,Entry procedure f,|A|≤1, Proceed to step (7),p≥0.5, Go to Step 8.

(6) Global search is carried out, random learning is carried out according to formula (12) to update the location of individuals with poor locations, and further update the location of individuals according to formula (9).

(7) Carry out the phase of encircling the prey, and update the individual position according to formula (10).

(8) Conduct the spiral bubble net predation stage, and update individual positions according to equation (11).

(9) Determine whether the iteration termination condition is met. If the condition is met, output the global optimal solution and location information; otherwise, proceed to step (3) to continue.The flow chart of MSWOA algorithm is shown in Fig 3.

## 4 Algorithm performance test

MATLAB R2022b programming software was used to evaluate the performance of the algorithm. To validate the effectiveness of the Mixed-Strategy Whale Optimization Algorithm (MSWOA), 12 benchmark functions were selected for testing. Among these, F1–F6 are unimodal functions, F7–F10 are complex multimodal functions, and F11 and F12 represent fixed-dimensional functions. Testing with a diverse set of functions effectively assesses the optimization performance of the algorithm. The details of these functions are provided in Table 1.

**Table 1:** Benchmark functions.

Function	Formula	Dimensionality	Radius	Optimal value
F1	$f_{x}=\sum_{i=1}^{n}X_{i}^{2}$	30	[-100,100]	0
F2	$f_{x}=\sum^{n}\left|x_{i}\right|+\prod {\mathrm{\backslash nolimits}}_{i=1}^{n}\left|x_{i}\right|$	30	[-10,10]	0
F3	$f_{x}=\sum_{i=1}^{n}{\left(\sum {\mathrm{\backslash nolimits}}_{j=1}^{i}x_{j}\right)}^{2}$	30	[-100,100]	0
_F4_	$f_{x}=\max_{i}\{\left|x_{i}\right|,1\leq i\leq n\}$	30	[-100,100]	0
F5	$f_{x}=\sum_{i=1}^{n-1}\left[100{\left(x_{i+1}-x_{i}^{2}\right)}^{2}+{\left(x_{i}-1\right)}^{2}\right]$	30	[-30,30]	0
F6	$f_{x}=\sum_{n}^{n}ix_{i}^{4}+\text{random}[0,1)$	30	[-1.28,1.28]	0
F7	$f_{x}=\sum_{i=1}^{n}-x_{i}\sin(\sqrt{\left|x_{i}\right|})$	30	[-500,500]	-12570
F8	$f_{x}=\sum_{i=1}^{n}\left[x_{i}^{2}-10\cos(2\;\;\;\pi \;\;\;x_{i})+10\right]$	30	[-5.12,5.12]	O
_F9_	$f_{x}=-20\exp(-0.2\sqrt{\frac{1}{n}\sum_{i=1}^{n}x_{i}^{2}}-\exp(\frac{1}{n}\sum_{i=1}^{n}\cos(2\;\;\;\pi \;\;\;x_{i}))+20+e$	30	[-32,32]	O
F10	$f_{x}=\frac{1}{4000}\sum_{i=1}^{n}x_{i}^{2}-\prod {\mathrm{\backslash nolimits}}_{i=1}^{n}\cos(\frac{x_{i}}{\sqrt{i}})+1$	30	[-600,600]	O
F11	$f_{x}=(\frac{1}{500}+\sum_{j=1}^{25}\frac{1}{j+\sum_{i=1}^{2}{\left(x_{i}-a_{ij}\right)}^{6}}{)}^{-1}$	30	[-65,65]	1
F12	$f_{x}={\sum_{i=1}^{11}\left[a_{i}-\frac{x_{i}(b_{i}+b_{i}x_{2})}{b_{i}^{2}+b_{i}x_{3}+x_{4}}\right]}^{2}$	30	[-5,5]	0.0003

The performance of the algorithm mentioned above is tested through two aspects. (1) Compare the convergence speed and accuracy of MSWOA, WOA(whale optimization algorithm), BOA(Butterfly optimization algorithm), GWO(Gray Wolf optimization algorithm), GAS(gravity search algorithm), PSO(particle swarm algorithm), and ABC(artificial bee colony algorithm) on the benchmark test function. Based on the same population size, maximum number of iterations and number of runs; (2) Analyze the test of the influence of different strategies on algorithm performance, based on independent tests under different improvement strategies.

### 4.1 Performance comparison with other optimization algorithms

To validate the performance of the proposed algorithm against other algorithms, the experimental setup includes a population size of 30 and a maximum of 500 iterations. Each algorithm is independently run 30 times to ensure statistical reliability. The mean value of the optimal simulation results and the standard deviation (std.dev) are used as performance evaluation metrics to compare the algorithms.

As shown in Table 2, by comparing the evaluation metrics of the optimal value and the average value, the MSWOA algorithm demonstrates the best optimization performance on the benchmark functions F1–F10 among the 12 test functions. For the benchmark function F11, the optimization accuracy of MSWOA is slightly inferior to that of the BOA and ABC algorithms, but it still outperforms other algorithms. In the case of F12, MSWOA’s accuracy is only marginally lower than that of the BOA algorithm.When using the standard deviation of the optimal value as the comparison metric, MSWOA exhibits slightly lower stability than the BOA, GWO, and ABC algorithms on benchmark function F5, and is less stable than BOA and ABC on function F11. However, overall, MSWOA still achieves the best performance in terms of stability across the majority of test functions.

**Table 2:** Comparison results of MSWOA and other optimization algorithms.

Reference function	Index	MSWOA	WOA	BOA	GWO	GSA	PSO	ABC
F1	mean std.dev	0 0	9.25 × 10^-70^ 4.04 × 10^-4^	1.30 × 10^-1^ 9.41 × 10^-13^	2.11 × 10^-23^ 3.00 × 10^-27^	8.12 × 10^-3^ 4.37 × 10^-2^	3.54 × 10^-4^ 1.07 × 10^-4^	1.27 × 10^2^ 6.75 × 10^2^
F2	mean std.dev	0 0	3.70 × 10^-50^ 1.80 × 10^-50^	4.46 × 10^-9^ 1.30 × 10^-9^	1.01 × 10^-16^ 7.34 × 10^-17^	6.07 × 10^-2^ 1.48 × 10^0^	1.99 × 10^-2^ 1.43 × 10^-2^	5.85 × 10^1^ 2.55 × 10^1^
F3	mean std.dev	0 0	4.29 × 10^1^ 1.45 × 10^4^	1.27 × 10^-11^ 1.03 × 10^-12^	1.33 × 10^-5^ 2.71 × 10^-5^	1.02 × 10^3^ 2.10 × 10^2^	8.99 × 10^1^ 2.76 × 10^1^	6.65 × 10^1^ 1.34 × 10^4^
F4	mean std.dev	0 0	4.99 × 10^1^ 2.80 × 10^1^	6.12 × 10^-12^ 3.86 × 10^-10^	1.08 × 10^-6^ 1.14 × 10^-6^	6.59 1.56	1.11 2.05 × 10^-1^	6.52 × 10^1^ 3.95
F5	mean std.dev	1.03 × 10^1^ 1.32 × 10^1^	2.80 × 10^1^ 4.49 × 10^-1^	2.90 × 10^1^ 2.71 × 10^-2^	2.72 × 10^1^ 7.10 × 10^-1^	8.19 × 10^1^ 6.43 × 10^1^	7.29 × 10^1^ 5.08 × 10^1^	2.97 1.52 × 10^6^
F6	mean std.dev	1.66 × 10^-4^ 2.45 × 10^-4^	2.86 × 10^-3^ 3.16 × 10^-2^	1.23 × 10^-3^ 5.79 × 10^-4^	1.90 × 10^-3^ 8.05 × 10^-5^	2.18 × 10^1^ 2.35 × 10^-5^	1.69 × 10^-1^ 5.41 × 10^1^	1.38 5.54 × 10^-1^
F7	mean std.dev	1.26 × 10^2^ 1.74	1.08 × 10^4^ 1.63 × 10^2^	---- ----	5.80 × 10^3^ 8.64 × 10^3^	-2.66 × 10^-3^ 3.56 × 10^2^	4.72 × 10^3^ 1.23 × 10^3^	4.71 × 10^60^ 2.19 × 10^61^
F8	mean std.dev	0 0	7.67 × 10^-15^ 2.43 × 10^-14^	6.50 3.50 × 10^1^	3.30 3.87	3.49 × 10^1^ 5.52	5.63 × 10^1^ 1.22 × 10^1^	2.51 × 10^2^ 1.34 × 10^1^
F9	mean std.dev	8.86 × 10^-16^ 9.86 × 10^-32^	4.20 × 10^-15^ 2.20 × 10^-15^	6.11 × 10^-9^ 4.07 × 10^-9^	1.03 × 10^-13^ 1.51 × 10^-14^	1.23 × 10^-8^ 3.30 × 10^-9^	1.65 × 10^-1^ 3.90 × 10^-1^	7.42 9.36E-01
F10	mean std.dev	0 0	9.96 × 10^-3^ 3.92 × 10^-3^	5.81 × 10^-12^ 3.25 × 10^-12^	2.56 × 10^-3^ 6.03 × 10^-3^	2.75 × 10^-1^ 5.90	1.10 × 10^-2^ 1.30 × 10^-2^	2.14 4.44 × 10^1^
F11	mean std.dev	1.45 9.80 × 10^-1^	3.80 3.31	1.27 5.31 × 10^-1^	4.75E 4.17	5.08 3.52	2.91 × 10^2^ 2.26	1.00 5.59 × 10^-1^
F12	mean std.dev	4.20 × 10^-4^ 1.38 × 10^4^	6.96 × 10^-4^ 4,53 × 10^-4^	3.80 × 10^-4^ 7.04 × 10^-5^	1.85 × 10^-4^ 4.96 × 10^-4^	8.50 × 10^-3^ 3.71 × 10^3^	9.15 × 10^-4^ 2.43 × 10^-4^	1.12 × 10^-3^ 1.75 × 10^-3^

**Fig 4:**
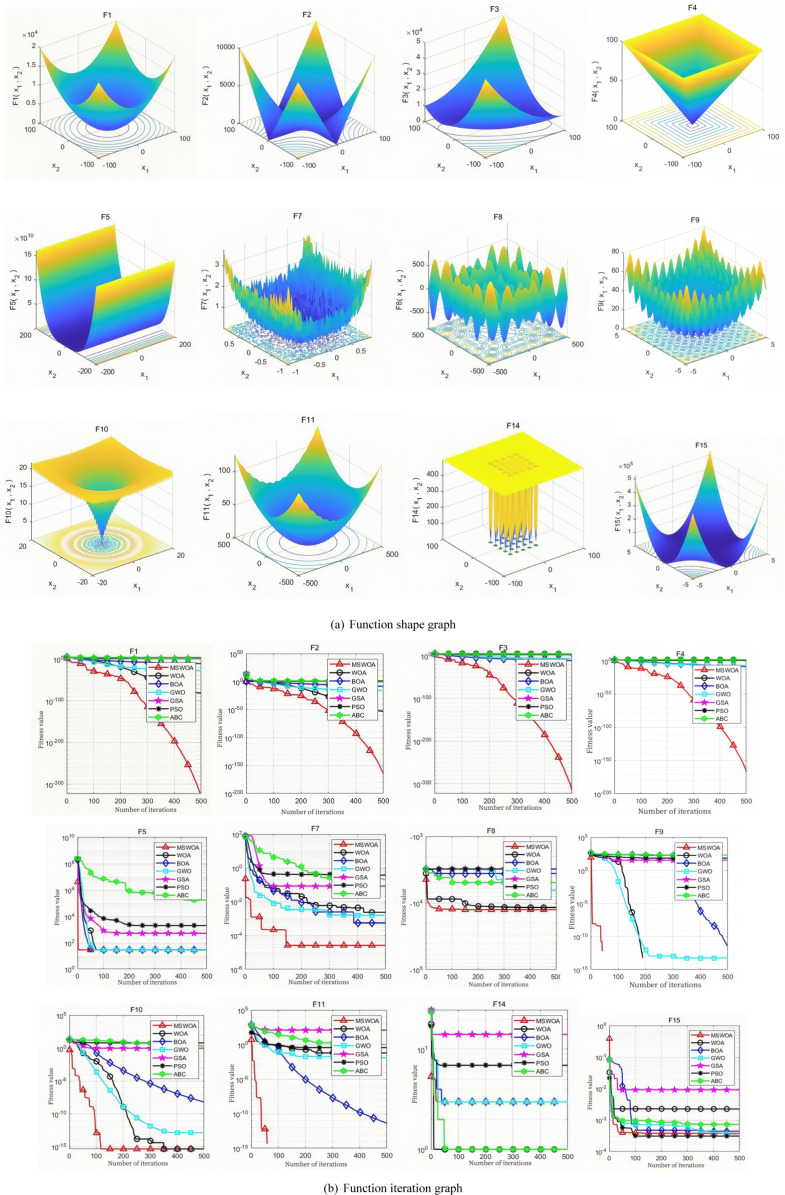
Function testing and iterative variation diagram.

By observing the convergence curve shown in Fig 4, we can directly assess the convergence speed of the proposed hybrid-strategy improved Whale Optimization Algorithm (MSWOA) and its enhanced ability to escape local optima. When the MSWOA algorithm performs optimization calculations on functions F1, F3, F8, F9, and F10, the use of the Sobol sequence for population initialization ensures a uniform distribution of the population. This increases the number of population individuals near the optimal solution, enabling the algorithm to find the optimal solution with the fewest iterations. As a result, MSWOA demonstrates strong optimization capabilities across all 12 benchmark functions. This is reflected in the convergence curve of MSWOA, which appears almost linear in the figure.In the optimization of F5 and F6, the presence of multiple inflection points in the convergence curve indicates the algorithm’s improved ability to escape local optima, further validating the effectiveness of the proposed hybrid strategy.

### 4.2 The effect of different strategies on algorithm performance is analyzed

In this paper, the proposed improvements to the Whale Optimization Algorithm (WOA) include Sobol sequence initialization, a nonlinear time-varying factor, adaptive weights, a Cauchy mutation strategy, and a stochastic learning strategy. To evaluate the impact of each strategy on the Mixed-Strategy Whale Optimization Algorithm (MSWOA), the population size is set to 30, the maximum number of iterations is 500, and each algorithm is independently run 30 times. The optimization is performed on the 12 benchmark functions listed in the table. The optimization results for each improvement strategy are presented in Table 3.

**Table 3:** Comparison results of different improved strategies.

Reference function	Index	WOA	WOA-1	WOA-2	WOA-3	WOA-4	WOA-5	MSWOA
F1	mean std.dev	9.25 × 10^-70^ 4.04 × 10^-70^	0 0	1.41 × 10^-72^ 4.38 × 10^-72^	0 0	5.71 × 10^-70^ 3.07 × 10^-69^	7.54 × 10^93^ 3.97 × 10^-92^	0 0
F2	mean std.dev	3.70 × 10^-50^ 1.80 × 10^-50^	0 0	4.46 × 10^-48^ 1.30 × 10^-47^	9.76 × 10^-170^ 0	3.70 × 10^-51^ 1.48 × 10^-50^	7.31 × 10^-62^ 2.74 × 10^-62^	0 0
F3	mean std.dev	4.29 × 10^1^ 1.45 × 10^4^	0 0	4.21 × 10^4^ 1.23 × 10^4^	1.68 × 10^-290^ 0	3.99 × 10^4^ 1.19 × 10^4^	3.17 × 10^3^ 7.76 × 10^3^	0 0
F4	mean std.dev	4.99 × 10^1^ 2.80 × 10^1^	0 0	4.12 × 10^1^ 2.59 × 10^1^	1.27 × 10^165^ 0	2.62 9.15	2.41 × 10^-15^ 7.55 × 10^-15^	0 0
F5	mean std.dev	2.80 × 10^1^ 4.49 × 10^-1^	2.50 × 10^1^ 8.15 × 10^-1^	2.90 × 10^1^ 4.46 × 10^-2^	2.80 × 10^1^ 2.83 × 10^-1^	2.29 × 10^1^ 4.06 × 10^1^	2.27 × 10^1^ 1.10 × 10^1^	1.03 × 10^1^ 1.31 × 10^1^
F6	mean std.dev	2.86 × 10^-3^ 3.16 × 10^-2^	9.36 × 10^-4^ 8.00 × 10^-4^	2.40 × 10^-3^ 1.78 × 10^-4^	7.92 × 10^-5^ 7.72 × 10^-5^	1.42 × 10^-3^ 2.85 × 10^-3^	8.92 × 10^-4^ 2.00 × 10^-3^	1.66 × 10^-4^ 2.47 × 10^-4^
F7	mean std.dev	1.08 × 10^4^ 1.63 × 10^2^	1.05 × 10^4^ 1.63 × 10^2^	1.08 × 10^4^ 1.80 × 10^3^	-1.25 × 10^3^ 2.00 × 10^3^	1.15 × 10^4^ 1.49 × 10^3^	1.23 × 10^3^ 1.67 × 10^3^	1.26 × 10^4^ 1.74
F8	mean std.dev	7.67 × 10^-15^ 2.43 × 10^-14^	0 0	6.50 3.50 × 10^1^	0 0	0 0	0 0	0 0
F9	mean std.dev	4.20 × 10^-15^ 2.20 × 10^-15^	8.88 × 10^-15^ 9.86 × 10^-32^	4.09 × 10^-9^ 2.21 × 10^-9^	8.88 × 10^-16^ 9.86 × 10^-32^	1.01 × 10^-15^ 6.38 × 10^-16^	2.07 × 10^-15^ 1.67 × 10^-15^	8.88 × 10^-16^ 9.86 × 10^-32^
F10	mean std.dev	9.96 × 10^-3^ 3.92 × 10^-3^	0 0	0 0	0 0	4.73 × 10^-8^ 2.12 × 10^-7^	0 0	0 0
F11	mean std.dev	3.80 3.31	2.35 8.67	2.57 2.56	1.49 8.00	2.12 1.99	2.96 2.92	1.45 9.80 × 10^-1^
F12	mean std.dev	6.96 × 10^-4^ 4,53 × 10^-4^	4.67 × 10^-4^ 2.59 × 10^-4^	6.59 × 10^-4^ 3.35 × 10^-5^	4.47 × 10^-4^ 9.99 × 10^-5^	6.67 × 10^-4^ 3.78 × 10^-4^	1.50 × 10^-3^ 1.78 × 10^-3^	4.21 × 10^-4^ 1.38 × 10^-4^

According to the data analysis in Table 3, among the five improvement strategies, the one that has the greatest impact on the performance of WOA algorithm is using Sobol sequence to initialize the population, followed by the adaptive strategy. In unimodal function, the random learning strategy is better than the Cauchy variation strategy with the best improvement in multi-modal function, while the improvement of nonlinear time-varying factors is worse than the other four. MSWOA, which combines five policy improvements, has better optimization accuracy and stability on most functions than WOA, which uses any of the strategy improvements.,However, each improvement strategy has verified the contribution of a single strategy. In WOA-1 and WOA-3, the Sobol sequence initialization population and the adaptive weight strategy were verified to have an impact on the algorithm. Changing the population distribution and enhancing the algorithm’s search ability had the greatest impact on the algorithm. The nonlinear time-varying factor in WOA-2 also had an influence on the convergence speed and performance of the algorithm. The Cauchy variation in WOA-4 significantly improved the algorithm’s efficiency. The random learning strategy in WOA-5 enhanced the algorithm’s global optimization performance. MSWOA algorithm combines the advantages of the five improved strategies. Under the same optimization accuracy, MSWOA converges faster, and the optimization accuracy of most test functions is higher than that of WOA algorithm with one improved strategy. Therefore, the feasibility of the five improved hybrid strategy algorithms is verified.

### 4.3 Statistical analysis of rank sum test of experimental results

**Fig 5:**
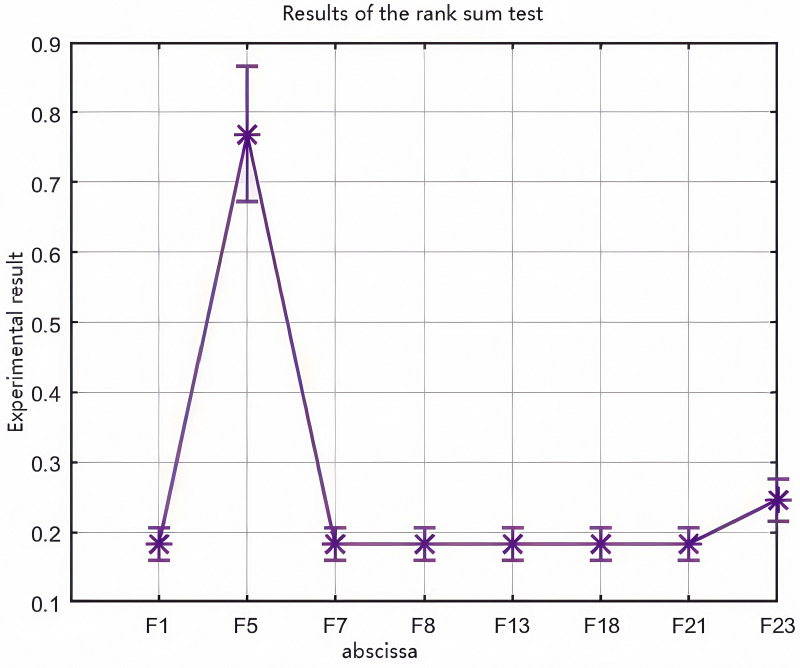
Rank sum data graph.

The rank sum test can be seen from the difference between statistical verification and JAYA, as can be seen from the data analysis in Fig 5. The improved WOA algorithm is quite different from the original one and meets the requirements of the improvement.

### 4.4 Test analysis

Through the above tests, it can be concluded first that the algorithm optimization performance and iteration speed of MSWOA are superior to those of other algorithms, and it is more stable. Based on the influence of different strategies on the original algorithm, it can be concluded that all five strategies have an impact on the original algorithm. Although a single strategy is not as effective as the mixed strategy, it has contributed to verifying the effectiveness of the strategies. After comparison, the MSWOA with the mixed improvement strategy has very strong performance. However, the complexity of MSWOA is more complex than that of other algorithms. The introduction of the five improvement strategies leads to additional operations, resulting in an increase in computational load, especially when dealing with large-scale problems, which may affect efficiency. However, this test experiment through Matlab testing did not affect the test process due to the increase in complexity. At the same time, attention should be paid to the adjustment of parameters. The mixed strategy increases the number of parameters and increases the difficulty of use. Therefore, we should be prepared to deal with the drawbacks of the algorithm, fully exert its advantages, and find ways to solve its shortcomings.

## 5 Path planning simulation test

### 5.1 Roblem description and environment modeling

When a fire occurs, the primary task of the algorithm is to analyze the internal environment of the underground building, avoid the fire source, locate safety exits, and plan the optimal evacuation route. To test the feasibility of the improved algorithm, the following assumptions about the environment are made:

Hypothesis 1: The underground building is simplified into a two-dimensional finite space. The positions of internal columns, walls, machinery, and stacked items (i.e., obstacles) are known, as are the locations of safety exits.

Hypothesis 2: Evacuees are treated as particles, ignoring individual differences. Their movement speed is constant, and the algorithm only influences their direction of movement.

Hypothesis 3: Obstacles and impassable fire zones after a mine fire are simplified into regular two-dimensional shapes to reduce computational complexity.

**Fig 6:**
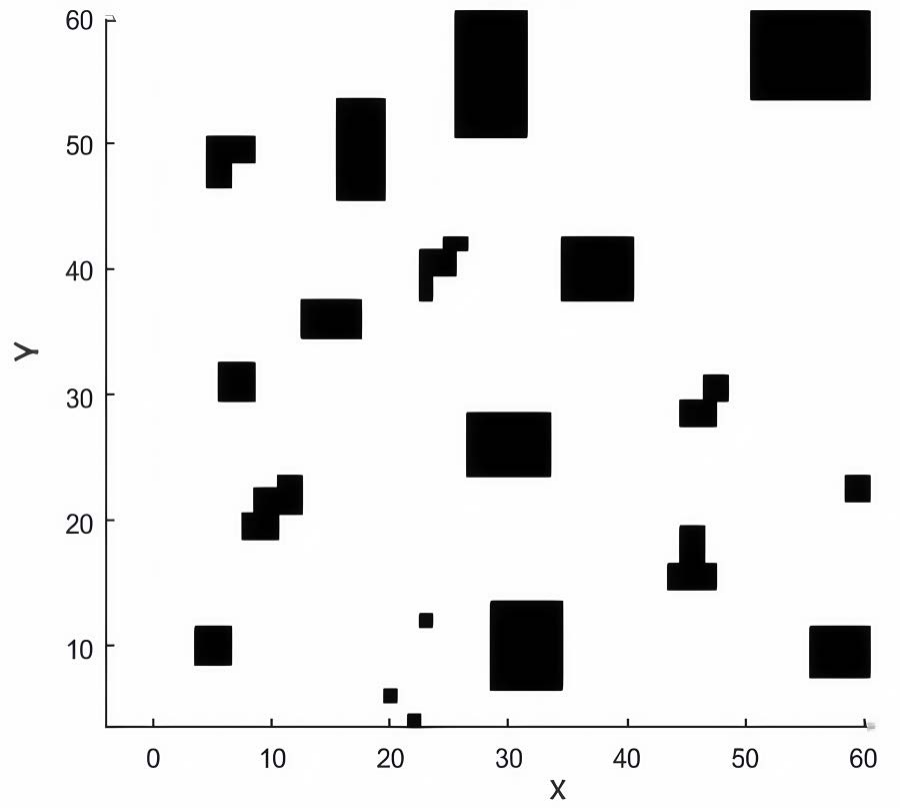
Map Environment model map.


The environment map shown in Fig 6 is generated by rasterizing the environmental information. The raster method is a traditional approach for path planning modeling [22]. A 60m × 60m simulation scene is constructed using this method. In the map, black grids represent obstacle areas, denoted by “1,” indicating impassable zones after a mine fire. White grids represent feasible areas, denoted by “0,” where evacuation paths can be planned. Each grid has a length of 1m and is numbered from bottom to top and left to right. Each grid has a unique sequence number and position coordinates, with the conversion formula as follows:

$$\begin{array}{c} {\{}_{y=a\left(NN+0.5-ceil\left(i/NN\right)\right)}^{x=a\left(mod\left(i,MM\right)-0.5\right)}\;\;; \end{array}$$ (15)

Among them,$x_{i}$ and $y_{i}$ is the coordinate of the position of the I-th grid;α represents the length of the side of the small grid, Usually take 1; MM and NN are the number of grids in row direction and column direction, respectively. mod is a redundant operator; ceil is rounded up.

### 5.2 Experimental verification and analysis

**Fig 7:**
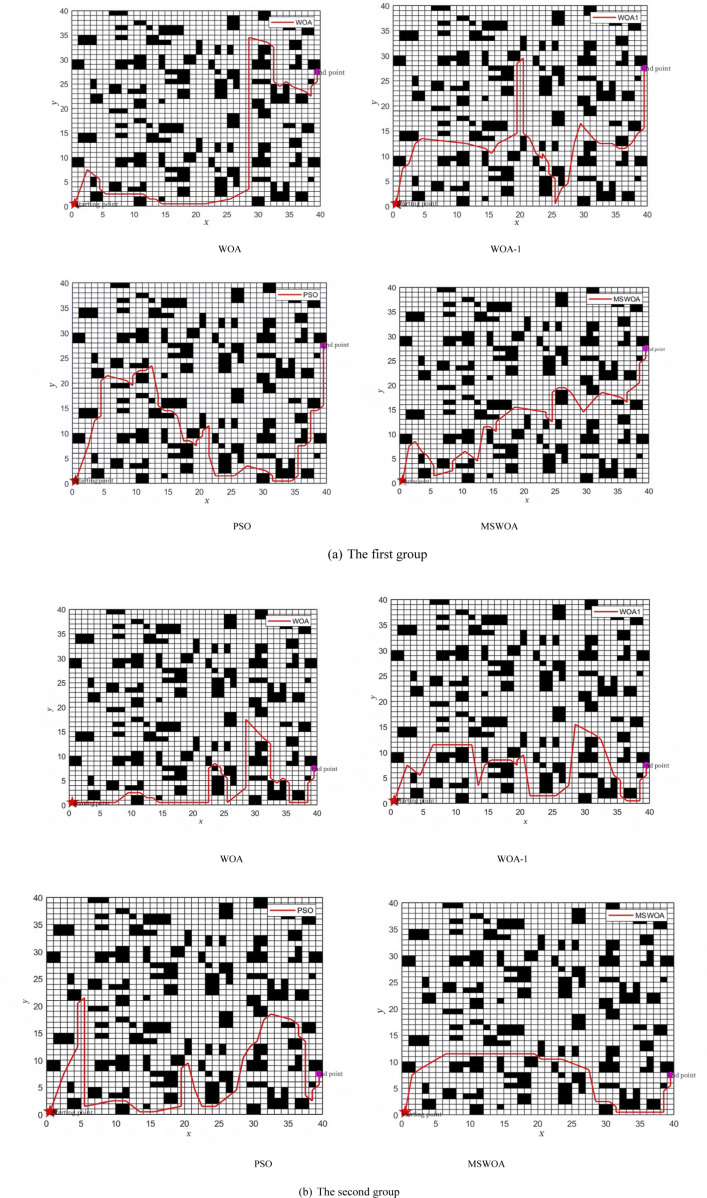
Simulation results of each algorithm path map.

**Fig 8:**
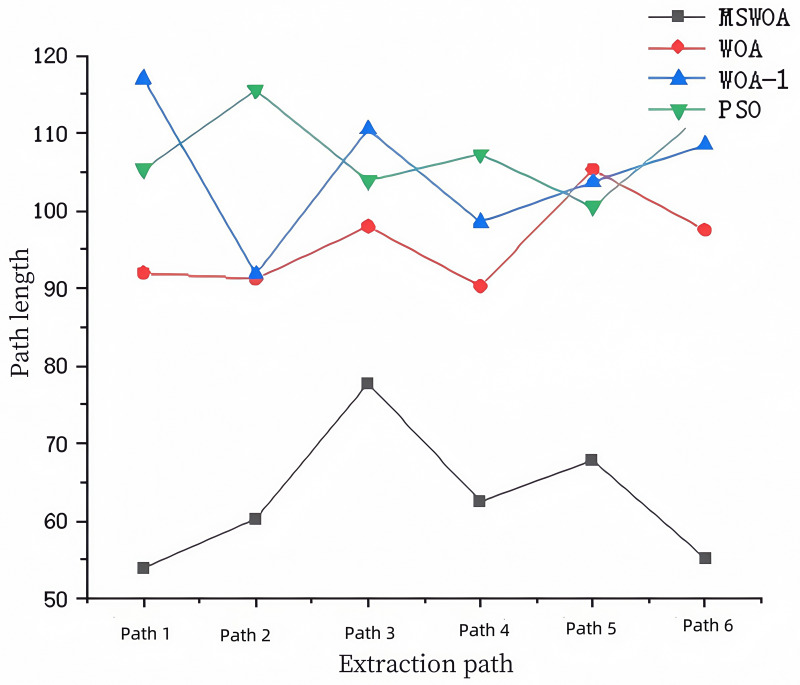
Path length variation diagram of each algorithm.

Based on the performance tests of the algorithm described above, the Mixed-Strategy Whale Optimization Algorithm (MSWOA) demonstrates outstanding performance after incorporating five improvement strategies. To further validate its effectiveness, the algorithm is applied to emergency path planning, specifically for planning escape routes after a mine fire in a grid-based environment. For comparison, the Whale Optimization Algorithm (WOA), WOA-1, and Particle Swarm Optimization (PSO) algorithms are also tested under the same conditions. This comparison aims to verify whether MSWOA maintains its superior performance in practical path planning scenarios.The path diagrams of the simulation results of each algorithm are shown in Fig 7; the diagrams of the changes in path lengths of each algorithm are shown in Fig 8.

Due to the inherent randomness of intelligent swarm algorithms, two path graphs are randomly selected for each algorithm to ensure a comprehensive analysis of their performance. To further validate the randomness and adaptability of the path planning, two different endpoints are used for testing. In Fig 7(a) and 7(b), the endpoints are consistent, allowing for a direct comparison between the two groups.From the perspective of path smoothness, as shown in Fig 7(a), when the endpoint is far from the starting point, the optimization accuracy of each algorithm varies significantly. The paths generated by WOA-1 and PSO exhibit recurring loops, resulting in unnecessarily lengthy routes. The WOA algorithm also shows similar issues between 30–40 meters. In contrast, the MSWOA algorithm plans a more direct and efficient path toward the target. Similarly, in Fig 7(b), when the endpoint is closer to the starting point, the paths planned by WOA-1 and PSO contain numerous sharp turns and large angles, leading to complex, lengthy, and less smooth routes. In comparison, the path generated by MSWOA is significantly smoother and more efficient. These observations clearly indicate that MSWOA outperforms the other algorithms in terms of optimization accuracy.

As shown in Fig 8, the path lengths generated by each algorithm can be directly compared. The path planned by MSWOA is significantly shorter. Specifically, the shortest path generated by MSWOA is 41.7% shorter than that of WOA, 42.3% shorter than WOA-1, and 48.5% shorter than PSO. On average, MSWOA’s paths are 32.2% shorter than WOA’s, 40.5% shorter than WOA-1’s, and 41.4% shorter than PSO’s. These results demonstrate that MSWOA can plan shorter and more optimized paths, highlighting its robustness and efficiency.

**Fig 9:**
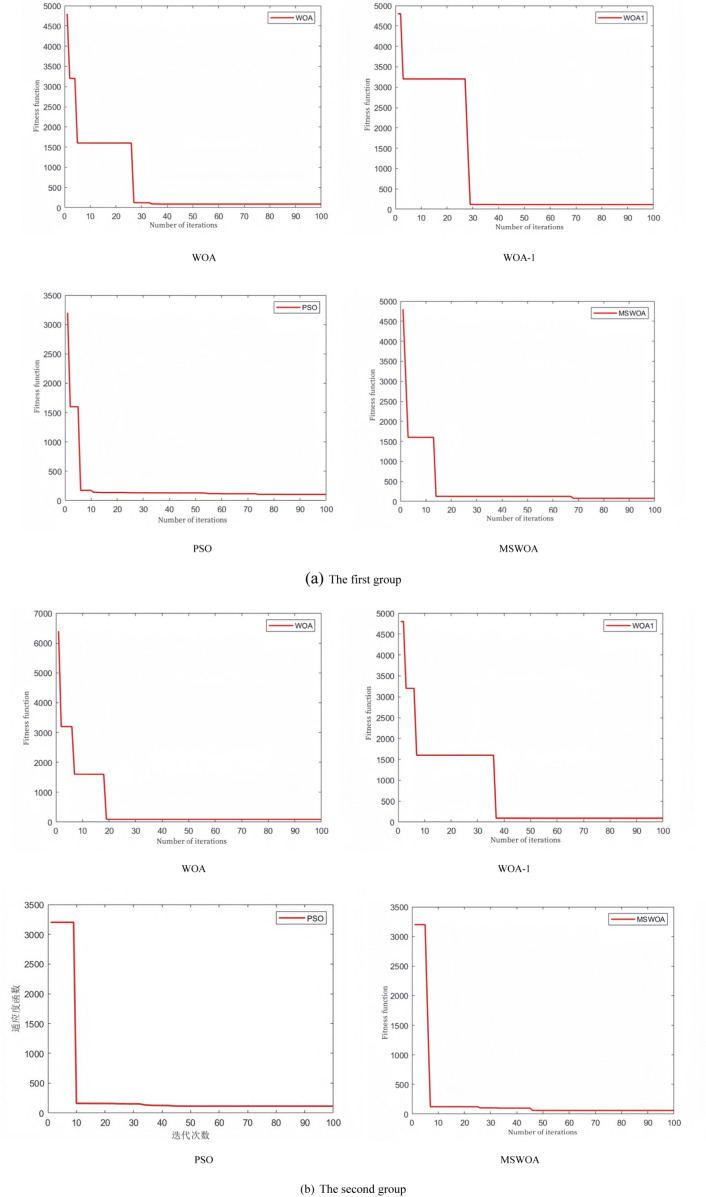
Algorithm iteration graph.

The algorithm iteration diagram is shown in Fig 9. MSWOA exhibits superior global search and local exploitation capabilities compared to WOA. Additionally, MSWOA demonstrates better optimization performance and the ability to escape local optima compared to WOA-1. In the early iterations, MSWOA initializes the population using the Sobol sequence, which ensures a uniform distribution of the population. Although this results in a slower initial convergence speed, it ultimately leads to better global optimization and faster convergence in the middle and late iterations compared to the other algorithms.

## 5.3 Experimental verification and analysis

Based on the analysis of the results after the path planning experiments, it can be known that MSWOA performed very well in the above experiments. The planned paths were more optimal, shorter and smoother. Compared with single strategies and other algorithms, the success of this algorithm improvement has also been confirmed. However, since this experiment was simulated, for the algorithm’s optimization performance in different environments, it still needs further research. Nevertheless, based on the existing experiments, the success of the MSWOA algorithm can be verified. It can provide better planning routes for the emergency rescue after mine fires. Future research should fully utilize the advantages of MSWOA and solve its disadvantages. Then it can be applied to robot or unmanned aerial vehicle for actual environment path planning. This has a very good research significance for the entire emergency aspect.

## 6 Conclusion

(1) Aiming at the problems of uneven initial population distribution, slow convergence speed, weak global search ability and easy to fall into local optimality, the standard whale algorithm introduced Sobol sequence to initialize the population and make the initial population evenly distributed; The global search capability is improved by nonlinear time-varying factor and inertia weight. The stochastic learning strategy is combined to increase the diversity of the population in the iterative process. Cauchy variation is introduced to improve the ability of the algorithm to jump out of local optimal.
(2) Compared with other intelligent algorithms and algorithms with only one improved strategy, MSWOA shows better optimization ability and fast convergence speed, and can find the shortest path in the case of unknown mine fire emergency.
(3) Through the path simulation diagram analysis, the path planned by MSWOA algorithm is significantly shorter. Compared with WOA algorithm, the shortest path is shortened by 41.7%, 42.3% and 48.5% compared with PSO algorithm. Compared with the average path length, the MSWOA algorithm is 32.2% shorter than the WOA algorithm, 40.5% shorter than the WOA-1 algorithm, and 41.4% shorter than the PSO algorithm. The shortest and smoothest path obtained by the MSWOA algorithm is analyzed by the path graph and the number of iterations graph. The MSWOA algorithm proposed in this paper has the best optimization effect, shortest path, better stability and robustness.
(4) This article has verified the success of the hybrid strategy improvement algorithm, and has also successfully demonstrated the effectiveness of the algorithm in path planning. At the same time, it has pointed out existing or potential problems, providing a research path for the escape and emergency issues in mine fires. In the future, when applied to robots or unmanned aerial vehicles, it is our outlook. The translation is as follows: This article has verified the success of the hybrid strategy improvement algorithm and has also successfully demonstrated the effectiveness of the algorithm in path planning. At the same time, it has pointed out existing or potential problems, providing a research path for the escape.
